# Epilepsy

**Published:** 1892-03

**Authors:** D. C. McLaren

**Affiliations:** Ottawa, Canada; Bureau of Clinical Medicine, I. H. A.


### EPILEPSY.

D. C. McLaren, M. D., Ottawa, Canada.

(Bureau of Clinical Medicine, I. H. A.)

The curability of a large proportion of cases of this distressing malady has been demonstrated in the past by Bœnninghausen, and though the wonderful success he attained seems to us younger Hahnemannians almost idealistic, it is yet full of encouragement to persevere and to make each defeat the stepping-stone for future victory. Two axioms of Constantine Hering's are of value in the treatment of this disease: the first is, to begin chronic diseases in general and all cases of epilepsy in particular with *Sulphur*, and secondly to guide the subsequent treatment according to the rubrics given in Bœnninghausen's hand-book, which now is, or ought to be, in the hands of every earnest homœopath.

The first case I have to report and my only definite success thus far with this formidable complaint is that of a young girl who suffered with epileptic seizures for a period of three or four years, from nine to thirteen. Her father died of cancer, showing very plainly the psoric condition causing her trouble. Early in 1890 she received one dose of *Belladonna* high, which gave five weeks' freedom from the constantly-recurring fits; upon their return she got *Calcarea*, which produced a strong aggravation lasting two weeks, followed by entire cessation for three months. The attacks returned in May, and for upwards of four weeks were battled with in vain, *Hyoscyamus* and *Cina* being given without result. At last, finding the head strongly drawn to the right side during the spasm, and noticing that as the attack wore off the whole right side, face, arm, and leg used to twitch, violently at first and then less so till all was quiet, and the aggravation during new and full moon, which had previously indicated *Calcarea*, being still present, all combined with the near approach of puberty, led to *Causticum*, which was given early in June, 1890. The trouble at once subsided, and a year has now elasped without a single recurrence of it. The girl has grown remarkably during this period; is now tall, strong, well-developed, and allowed to attend church, Sunday-school and day-school, which had all been denied her for some years.

While attending this case an elder sister, eighteen years of age, broke down and seemed almost at the point of death from anæmia, not having menstruated for three years, and being in a pitiful state of emaciation. She would sit hour after hour with her feet close to the stove and her head wrapped up in a shawl; when in bed she must have the bedclothes over her head. One administration of *Silicea* made an entire cure of this case, the girl being now reasonably well and strong.

I have also two other cases of epilepsy now under treatment, both boys, one eighteen, the other fourteen. The first one began with *Sulphur* on September 5th, 1890; was given *Calcarea* on November 19th, and *Belladonna* on January 24th, 1891, which last was followed by a slight aggravation. On February 23d he had an attack while asleep at night with unconscious urination; for this he got *Causticum*, which gave entire relief until April 10th, with another interval to May 3d, after which he received a dose of *Sulphur*. There is a satisfactory improvement in this case, though far from cured as yet.

The second boy is the son of a man who used to be a hard drinker, and though now a sober, steady man, has the mortification of seeing the results of his folly daily manifested in his unfortunate son. This case has been under treatment eight months without any noticeable improvement. The majority of attacks are at night, frequently accompanied with urination. He was under *Causticum* from January 31st to May 2d without result, and is now under *Calcarea* since May 24th, with what result remains yet to be seen, but the probabilities are in favor of long and troublesome treatment and much disappointment, though, I hope, ultimate success.

A fourth case, that of a married woman approaching the grand climacteric, has too recently come under treatment to furnish any material for reporting.

